# A Contemplated Blow at Our Horse-industry

**Published:** 1898-03

**Authors:** 


### EDITORIAL.

### A CONTEMPLATED BLOW AT OUR HORSE-INDUSTRY.

THE German Ambassador to this country corrects the report that his government is about to exclude American horses on the ground of their infection with influenza. He states that the Prussian Diet has directed an inquiry as to the possible dangers in the admission of these horses, and it would determine later the necessity for quarantine measures. We have no doubt that, as Germany has placed barriers against almost all our other leading export-products, our horses will come under the ban next. These conditions, incidental to the shipping of horses long journeys, are known in every horse-mart of the world, and are only of temporary moment, and we can only see the extension of the disposition to question American products. A little more inquiry as to German products sent to this country, and admitted without question, would do much to stop this offensive disposition toward the extension of American commerce, and the exclusion of any products that we can and do lead the world in producing.

